# Understanding the Rare Breast Cancer Subtypes With Variations in Histopathology, Molecular Profiling, Clinical Outcomes and Therapeutic Approaches

**DOI:** 10.7759/cureus.96952

**Published:** 2025-11-16

**Authors:** Mai Abdelgelil, Abdulraheem Alshangiti, Wiam Taha, Osama Soliman, Raja Aldandan, Ahmed Alabbas, Haider Abbas, Faisal Azam

**Affiliations:** 1 Department of Adult Medical Oncology, King Fahad Specialist Hospital, Dammam, SAU; 2 Department of Clinical Oncology, Assiut University Hospital, Assiut, EGY

**Keywords:** apocrine carcinoma, lymphoepithelioma-like carcinoma, metaplastic carcinoma, mucinous carcinoma, neuroendocrine breast cancer, rare breast cancer subtypes, triple negative breast cancers

## Abstract

Background

Breast cancer is a heterogeneous disease with significant variation in clinical behaviour, pathological morphology and molecular patterns. While most of the breast cancer cases follow common histological and clinical patterns, but a few rare subtypes behave differently with a difference in prognosis and response to therapy. Recognizing these subtypes of breast cancer is important for accurate diagnosis and treatment planning.

Aims and objectives

This study aimed to evaluate the clinical features, pathological criteria, and outcomes of patients with these rare breast cancer subtypes treated at King Fahad Specialist Hospital (KFSH-D), a biggest regional tertiary care center.

Patients and methods

This is a retrospective, observational, descriptive study analyzing 30 patients diagnosed with rare breast cancer subtypes and treated at KFSH-D between January 2019 and December 2024.

Results

We included 30 patients with rare pathological breast cancer subtypes. Among them, eight patients (27%) had apocrine carcinoma. The mean age at diagnosis for this group was between 55 and 60 years and a mean tumor size was greater than 2 cm. Additionally, 14 (46%) patients had metaplastic carcinoma, with a mean age at diagnosis of 45-60 years and tumor sizes ranging between 3 and 5 cm. Five (17%) patients were diagnosed with mucinous adenocarcinoma. Their mean age at diagnosis was 70 years, with tumors larger than 2 cm. All of these patients had stage N0-N1 disease and were positive for estrogen receptor (ER), progesterone receptor (PR), and negative for Her-2/neu on immunohistochemistry (IHC). One (3%) patient had lymphoepithelioma-like carcinoma. She was diagnosed at 51 years of age, with a tumor size of 2.2x3.2 cm. She underwent simple mastectomy and sentinel lymph node biopsy (SLNB). The tumor was histologic grade III with no lymphovascular invasion or intraductal component. We also identified two (7%) patients with invasive ductal carcinoma (IDC) showing neuroendocrine differentiation. Both were positive for chromogranin A and synaptophysin on IHC. Their ages at diagnosis were 55-65 years, and tumor sizes ranged from 4 to 6 cm. These tumors were histologic grade III, hormone receptor-positive, and Her-2/neu negative. Survival outcomes varied across groups, with mucinous carcinoma showing the most favorable prognosis.

Conclusion

Rare subtypes of invasive breast cancers have distinct clinical and pathological behaviours with different outcomes. Recognition of these tumors will help in patient-tailored treatment strategies and will contribute to better patient management.

## Introduction

There has been a lack of focus on recognizing the special types of breast cancers that, due to their distinct morphology, molecular characteristics, and clinical features, might display prognostic and predictive characteristics that vary from those of invasive ductal carcinoma (IDC) of no special type (NST) or not otherwise specified (NOS) [1]. IDC accounts for approximately 60%-75% of all breast cancer cases, while special types of breast cancer make up the remaining 25%. According to the most recent edition of the World Health Organization (WHO) classification, there are 17 different histological subtypes including invasive lobular carcinoma, tubular carcinoma, invasive cribriform carcinoma, mucinous carcinoma, other mucin-rich tumors, medullary carcinoma, metaplastic carcinomas, neuroendocrine tumors, apocrine carcinoma, adenoid cystic carcinoma, invasive papillary carcinoma, invasive micropapillary carcinoma, secretory carcinoma, among other less common types [2].

Evidence from the literature suggests that these special types of breast cancer may have different outcomes compared to IDC, even when matched for similar biological features and disease stages [3] and so identifying these special subtypes are important. Some of those subtypes have an excellent prognosis. Therefore, accurate and reliable histopathological evaluation is essential to detect these unique cancer types and appropriately determine the need for adjuvant therapy [1,4].

Our study aims to explore the unique characteristics of these rare breast cancer subtypes, highlighting their histological, molecular, and clinical features in order to propose an appropriate treatment approach.

## Materials and methods

This is a retrospective, descriptive analysis of 30 patients diagnosed with uncommon histological subtypes of breast cancer and managed at King Fahad Specialist Hospital, a tertiary referral oncology center in the Eastern Province of Saudi Arabia.

Study period and participants

Our study included patients diagnosed and treated for rare histological subtypes of breast cancer between January 2019 and December 2024. We have used the World Health Organization (WHO) classification to define and include patients with rare subtype of invasive breast carcinoma in our study.

Rare subtypes in our study were defined as invasive breast carcinomas that account for <5% of all breast cancers. These included: apocrine carcinoma, metaplastic carcinoma, mucinous (colloid) carcinoma, lymphoepithelioma-like carcinoma, and invasive ductal carcinoma with neuroendocrine differentiation.

Patient data was collected from the hospital management information system and the institutional cancer registry after approval from the local Institutional Review Board (IRB protocol number ONC0419). We collected information on certain variables like demographic information (age, sex, menopausal status), tumor characteristics (size, grade, stage, nodal status), receptor status (estrogen receptor (ER), progesterone receptor (PR), human epidermal growth factor receptor 2 (HER2)), immunohistochemistry (IHC) markers where available like chromogranin A, synaptophysin, CK7, CD117, type of surgery (e.g., breast-conserving surgery, mastectomy, sentinel lymph node biopsy, axillary clearance), type of systemic treatments and radiotherapy received (e.g., chemotherapy, endocrine therapy, targeted therapy), in addition to the clinical outcomes, including disease-free survival (DFS), progression-free survival (PFS), and overall survival (OS).

All IHC studies were performed in our hospital by our pathology department following institutional protocols. ER, PR, and HER-2 results were interpreted based on standard scoring guidelines, with additional markers (chromogranin A, synaptophysin, GATA3, CK7, CD117, vimentin), which were used as appropriate for subtype classification. We used a descriptive statistical approach. Continuous variables such as age and tumor size were expressed as means or ranges, while categorical variables such as receptor status and stage were reported as frequencies and percentages. Survival outcomes were estimated using available follow-up data.

## Results

Among the 30 patients with rare pathological subtypes of breast cancer included in this retrospective analysis, eight patients (~27%) were diagnosed with apocrine carcinoma. The mean age of patients with apocrine carcinoma was 57 years (range, 55-60 years), with a mean tumor size >2 cm. Of these, five had N1 nodal involvement and three had N0 disease. All patients exhibited triple-negative breast cancer (TNBC) biology. Six patients with stage I/II disease underwent breast-conserving surgery followed by adjuvant radiotherapy and chemotherapy. The remaining two patients presented with de novo metastatic disease and demonstrated a favorable response to platinum-based chemotherapy, in line with standard treatment protocols for TNBC. The median OS in this group was approximately 24 months (range 18-30 months).

Fourteen (46%) out of 30 patients had metaplastic carcinoma with a mean age at presentation of 51 years (range, 45-60 years), with tumor sizes between 3 and 5 cm. Ten patients had node-negative disease, while four presented with N1-N2 nodal involvement. All exhibited triple-negative biology. Twelve patients with stage II/III disease underwent mastectomy after neoadjuvant chemotherapy due to a large tumor burden. Two patients had upfront metastatic disease and received platinum-based chemotherapy, with a median PFS of 18 months (range, 16-20 months).

Five (17%) patients had mucinous adenocarcinoma with a mean age at diagnosis of 70 years (range,65-75 years). The mean tumor size was >2 cm. All had N0-N1 disease and demonstrated ER and PR positivity with HER2-negative status. These patients underwent breast-conserving surgery with axillary staging using sentinel lymph node biopsy (SLNB). While only two received adjuvant radiotherapy, all were treated with adjuvant hormonal therapy. The median OS in this group was approximately 36 months.

One patient (~3%) was diagnosed with lymphoepithelioma-like carcinoma. She was 51 years old at diagnosis, with a tumor size of 2.2×3×2 cm. She underwent a simple mastectomy with SLNB. The tumor was histologic grade III, with no lymphovascular invasion or intraductal component. IHC analysis revealed expression of E-cadherin, cytokeratin 7 (CK7), and CD117 in tumor cells. CAM 5.2 was patchy positive, GATA3 was focally and weakly positive in epithelioid cells, while ER, PR, and HER2 were negative. Vimentin was positive only in stromal and lymphoid elements. All sentinel lymph nodes were negative. She completed six cycles of paclitaxel and carboplatin chemotherapy followed by radiotherapy. She is alive at ~30 months of follow-up.

Finally, two patients (7%) were diagnosed with IDC with neuroendocrine differentiation. IHC confirmed positivity for chromogranin A and synaptophysin. These patients were between 55 and 65 years old, with tumor sizes ranging from 4 to 6 cm. Both tumors were histologic grade III, hormone receptor-positive, and HER2-negative. They presented with stage II/III disease and underwent mastectomy and SLNB following neoadjuvant chemotherapy. Postoperatively, both received adjuvant hormonal therapy in combination with abemaciclib. Both patients have remained disease-free for over three years.

Table 1 shows the discrepancy between such rare breast cancer subtypes in patients’ demographics and tumor characteristics.

**Table 1:** Patients’ demographics and tumor characteristics TNBC: Triple-negative breast cancer.

Subtype	Number of Patients (%)	Mean Age in Years(range)	Mean Tumor Size (cm)	Nodal Involvement	Receptor Status
Apocrine Carcinoma	8 (26.7%)	57 (55-60)	>2	N1 (5), N0 (3)	TNBC
Metaplastic Carcinoma	14 (46.7%)	51 (45-60)	3-5	N0 (10), N1-N2 (4)	TNBC
Mucinous Adenocarcinoma	5 (16.7%)	70 (65-75)	>2	N0-N1 (5)	ER/PR+, HER2-
Lymphoepithelioma-like Carcinoma	1 (3.3%)	51	2.2 × 3 × 2	N0 (1)	TNBC
IDC with Neuroendocrine Differentiation	2 (6.7%)	60 (55-65)	4-6	-	ER/PR+, HER2-

Moreover, the differential survival between these rare subtypes is shown in Table 2.

**Table 2:** Outcomes of different treatment modalities used for management of rare breast cancer subtypes. OS: overall survival, PFS: progression-free survival, NR: not reached.

Subtype	Number of patients	Median OS in months (range)	Median PFS in months(range)
Apocrine Carcinoma	8	~24 (18-30)	NR
Metaplastic Carcinoma	14	NR	~18 (16-20)
Mucinous Adenocarcinoma	5	~36 (34-38)	NR
Lymphoepithelioma-like Carcinoma	1	~30 (28-32)	NR
IDC with Neuroendocrine Differentiation	2	NR	NR

## Discussion

Rare types of breast cancer are a heterogeneous group of cancers. It comprises 5%-10% of TNBCs [5]. The study of rare breast cancer subtypes has been limited by several confounding factors, including small sample size, overlapping diagnostic categories and variability in the interpretation of histopathologies. Knowledge of these rare subtypes holds promise for advancing our knowledge and devising more appropriate treatments. Here, we review the molecular and clinical features and treatment of more commonly encountered rare and unusual forms of breast cancers.

Apocrine carcinoma constitutes approximately 0.4% [6,7] of invasive breast tumors and is often regarded as a variant of IDC or NST [8]. O'Malley and Bane [8] cite two studies [9,10] that compared invasive apocrine carcinomas with matched NST tumors and found no difference in survival outcomes. These studies concluded that apocrine carcinoma does not represent a clinically distinct subtype. However, more recently, Japaze et al. [11] argued that pure invasive apocrine carcinoma is a distinct disease, which, when strictly defined by their criteria may be less aggressive than IDC and NST. Vranic et al. [12] and Weigelt et al. [13] proposed that these cancers have heterogeneous gene-expression profiles with multiple molecular subtypes rather than constituting a distinct category; however, they noted that pure apocrine cancer, limited to a characteristic profile of ER negative and PR negative defines a group that is either HER-2 overexpressing or triple-negative with consequent therapeutic implications. In our study, all patients with apocrine pathology were triple negative, treated with standard treatment protocol for TNBC with IDC pathology, achieving good survival benefit.

The incidence of metaplastic breast carcinoma (MeBC) is approximately 0.2%-0.6% [14,15-17], with a median age at presentation of 47-61 years [14,15-17,18-20]. A Japanese study [19] compared cases of IDC (n=6,137), lobular breast cancer (n=301), and MeBC (n=46). In this study, there was a notable decrease in both lymph node involvement and the use of neoadjuvant or adjuvant therapies in the MeBC group, although a higher frequency of skin invasion than in patients with IDC. On the other side, a Korean database study (n=35) found that patients with MeBC had larger tumors, less nodal involvement and were more likely to have mastectomy and chemotherapy. They were less likely to have adjuvant radiation than patients with IDC [16]. This data matches that of our study. The mean age at presentation of our patients was 45-60 years with a mean tumor size between 3 and 5 cm. Seventy-one percent of patients had lymph node-negative disease and 86% of them were treated with mastectomy after neoadjuvant chemotherapy.

Mucinous (colloid) carcinoma is another special-type carcinoma that predicts an excellent prognosis when present in pure form [21,22]. Pure mucinous carcinoma accounts for 1%-4% of all breast cancers. It is generally diagnosed at an advanced age. In a retrospective series of 11,400 cases of pure mucinous carcinoma, the median age at diagnosis was 71 years versus 61 years observed in patients with infiltrative ductal carcinomas [23]. The majority of these lesions are well-differentiated HR-positive and HER2-negative. The axillary lymph nodes are rarely involved. These features account for the favorable prognosis of this breast cancer subtype, with a five-year breast cancer-specific survival rate of 94% compared with 82% of the infiltrating ductal carcinoma counterpart. Our study supports the medical literature. Our patients' mean age was 70 years with a mean tumor size of >2 cm, all had N0 (lymph node negative)-N1 (lymph node positive) disease, and ER- and PR-positive and HER-2 negative tumor biology.

Lymphoepithelioma-like carcinoma (LELC) is an undifferentiated carcinoma with lymphocytic infiltration between tumor cells. In 1994, Kumar and Kumar diagnosed the first breast carcinoma case with the histopathologic features of LELC [24,25]. It is believed that most LELCs are associated with Epstein-Barr virus (EBV), but none of the published cases of this rare subtype was EBV-positive [26] and mostly had a good prognosis [27]. Since LELC is an uncommon tumor type, the diagnosis may be challenging due to the similarity of histologic features with invasive ductal or lobular lymphocytic invasive carcinoma. Our study also reported a case of an early-stage LELC with triple-negative biology. She responded well to the standard treatment protocol with an OS of 30 months.

Another rare breast cancer subtype is a neuroendocrine carcinoma. It is most frequently a small cell carcinoma. As per the WHO figures, neuroendocrine breast cancer occurs in <1% of patients, but breast cancer with neuroendocrine differentiation is comparatively more common (10%-18%) [28].

Given the growing interest in the viral etiology of rare breast cancer subtypes, including reports of human papillomavirus (HPV) DNA detection in some cases, preventive strategies such as HPV vaccination that have proven effective in reducing cervical cancer incidence may have broader implications that merit further study [29].

This study has potential limitations. As our study is retrospective with a small sample size, we still need more conclusive research to identify more about those rare breast cancer subtypes.

## Conclusions

Special rare histological subtypes of breast cancer encompass a broad clinical spectrum, ranging from patients with indolent disease, where there is limited evidence to justify the routine use of endocrine therapy to those with higher-risk features, for whom combined modality treatments are recommended. Certain breast cancer subtypes have a favorable outcome - such as tubular, cribriform, mucinous, and papillary carcinoma. These can be adequately managed with either no systemic therapy or endocrine therapy alone, reflecting an excellent prognosis. Conversely, treatment approaches for invasive lobular carcinoma (ILC) and apocrine carcinoma need to be individualized. Biological characteristics of the tumor and the extent of disease should be considered in their treatment, which is similar to invasive ductal carcinoma (IDC).

The triple-negative breast cancer (TNBC) was considered a homogeneous group, but now it is recognized to comprise different histological subtypes with variable prognoses and treatment implications. For example, adenoid cystic and medullary carcinomas tend to have more favorable outcomes, whereas metaplastic carcinomas are typically more aggressive and refractory to standard therapies. This highlights the importance of histological subtyping even within traditionally defined molecular classes. Furthermore, genomic profiling of these rare histological variants may uncover distinct oncogenic pathways, potentially offering insights into mechanisms of disease progression and new treatment options.

In conclusion, a more detailed understanding of the clinical and genomic landscape of these rare breast cancer histologies is essential to advance personalized treatment approaches and optimize outcomes across the full spectrum of breast cancer.
